# An Investigation of Quorum Sensing Inhibitors against *Bacillus cereus* in The Endophytic Fungus *Pithomyces sacchari* of the *Laurencia* sp.

**DOI:** 10.3390/md22040161

**Published:** 2024-03-31

**Authors:** Shi-Liang Xiang, Kai-Zhong Xu, Lu-Jun Yin, Ai-Qun Jia

**Affiliations:** Key Laboratory of Tropical Biological Resources of Ministry of Education, School of Pharmaceutical Sciences, Hainan University, Haikou 570228, China

**Keywords:** *Laurencia* sp., endophytic fungus, *Pithomyces sacchari*, demethylincisterol A_3_, *Bacillus cereus*, quorum sensing

## Abstract

*Bacillus cereus*, a common food-borne pathogen, forms biofilms and generates virulence factors through a quorum sensing (QS) mechanism. In this study, six compounds (dankasterone A, demethylincisterol A_3_, zinnimidine, cyclo-(L-Val-L-Pro), cyclo-(L-Ile-L-Pro), and cyclo-(L-Leu-L-Pro)) were isolated from the endophytic fungus *Pithomyces sacchari* of the *Laurencia* sp. in the South China Sea. Among them, demethylincisterol A_3_, a sterol derivative, exhibited strong QS inhibitory activity against *B. cereus*. The QS inhibitory activity of demethylincisterol A_3_ was evaluated through experiments. The minimum inhibitory concentration (MIC) of demethylincisterol A_3_ against *B. cereus* was 6.25 μg/mL. At sub-MIC concentrations, it significantly decreased biofilm formation, hindered mobility, and diminished the production of protease and hemolysin activity. Moreover, RT-qPCR results demonstrated that demethylincisterol A_3_ markedly inhibited the expression of QS-related genes (*plcR* and *papR*) in *B. cereus*. The exposure to demethylincisterol A3 resulted in the downregulation of genes (*comER*, *tasA*, *rpoN*, *sinR*, *codY*, *nheA*, *hblD*, and *cytK*) associated with biofilm formation, mobility, and virulence factors. Hence, demethylincisterol A_3_ is a potentially effective compound in the pipeline of innovative antimicrobial therapies.

## 1. Introduction

Seaweeds, the largest group of oceanic plants, are capable of producing various metabolites, including polysaccharides, terpenes, and lectins [1]. Algal metabolites exhibit a range of beneficial effects, such as antibacterial, antiviral, antioxidant, and anticancer properties [1,2,3,4]. Endophytic fungi, residing within the plant body, coexist without harming the host and can synthesize compounds similar to those produced by the host plant [5]. For instance, the fungal endophyte *Taxomyces andreanae*, found within the inner bark of the *Taxus brevifolia* (Pacific yew tree), is capable of synthesizing paclitaxel derivatives [6]. Similarly, *Diaporthe longicolla*, an endophyte from the leaves of *Saraca asoca*, produces metabolites with noted antibacterial and antioxidant properties [7]. The ethyl acetate extract from *Nigrospora sphaerica*, another endophytic fungus, exhibits potent antioxidant activities, effectively combating free radicals [8]. Additionally, *Hyllosticta capitalensis*, an endophyte, is known for generating a variety of bioactive compounds that possess both antibacterial and antitumor potential [9]. Recent studies reveal that bioactive substances from endophytic fungi, known for their antioxidant, antibacterial, anti-inflammatory, and cancer cell inhibitory effects, have become a prominent focus for screening and research [10].

Foodborne diseases stemming from pathogens constitute a widespread global public health concern [11]. *Bacillus cereus* is a Gram-positive bacterium known for its ability to cause food poisoning [12]. Widely distributed in air, water, and soil, *B. cereus* may be present in various foods, including potatoes, rice, and beans [12]. *B. cereus* can produce enterotoxins and emetic toxins, leading to symptoms such as diarrhea and vomiting in affected individuals [13]. At the same time, it can form biofilms, enhancing its efficiency in producing harmful metabolites [14]. With the assistance of biofilms, *B. cereus* can evade the decomposition of digestive enzymes [15]. The flagella of *B. cereus* aid in movement to suitable locations and the formation of *B. cereus* biofilm is intricately connected to this locomotion process [16]. It can modulate gene expression through quorum sensing (QS), exercising control over biofilm formation, virulence factors, and spore production [17].

Given that the production of harmful toxins, as well as functions like mobility and membrane activity in *B. cereus*, rely on QS, addressing its impact on human health underscores the importance of developing treatments to disrupt its QS. Therefore, this study aimed to identify compounds from marine algal endophytic fungi that can inhibit the QS function of *B. cereus*, thereby attenuating its pathogenicity.

## 2. Results

### 2.1. Identification of Active Strains

**Figure 1:**
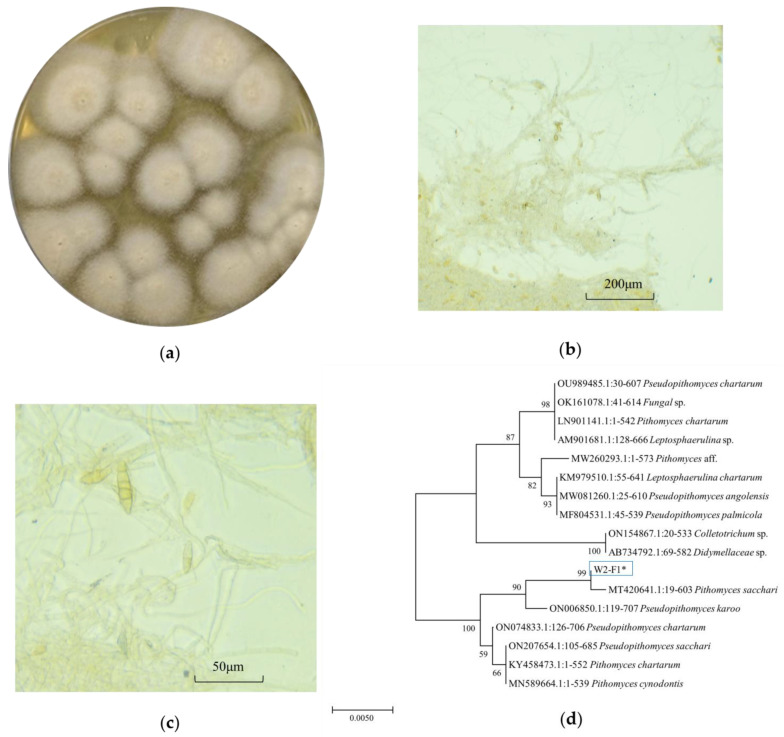
Morphological and phylogenetic tree of *P. sacchari* strain. (**a**) Colonies grown on a PDA. (**b**,**c**) The spores and mycelia were under a microscope with 40× and 100× magnification. (**d**) Phylogenetic tree.

Mycelium grown on potato dextrose agar (PDA) exhibited a white coloration and adopted a coarse and cotton-like texture. With the progression of growth, the central color could progressively transform to yellow (Figure 1a). The cells of the conidia exhibited microscopic features such as yellow coloration, oval or slightly oval shape, and 3–5 diaphragms (Figure 1b). The spore wall was smooth. The conidia were septate, elongated, and pale brown in color (Figure 1c). The strain was determined as *Pithomyces* by comparing its gene sequences to those in the gene library (Figure S9). The most closely related strain of W2-F1 was *Pithomyces sacchari* (99.00%) (Figure 1d).

### 2.2. The Elucidation of Compounds 

**Figure 2:**
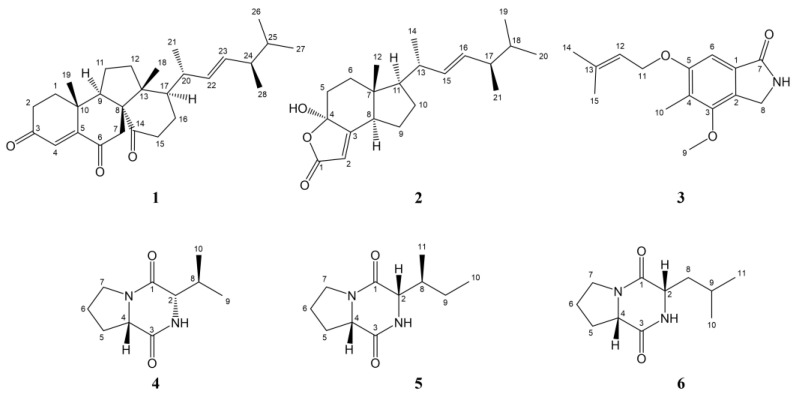
The chemical structures of the isolated six compounds (**1**–**6**).

The isolated six compounds were identified using nuclear magnetic resonance (NMR) and mass spectrum (MS) techniques, and their structures were determined by comparing them with the literature data (Figure 2). The NMR and MS spectra of the six compounds are shown in the Supporting Information (Figures S3–S8).

Compound **1** (4.8 mg) was identified as follows. Dankasterone A (C_28_H_40_O_3_, *m/z* = 425.3058 [M + H]^+^, cald. for 425.3050) [18]. ^1^H-NMR (400 MHz, MeOD) *δ* 6.16 (s, H-4, 1H), 5.35–5.31 (m, 2H), 2.87 (td, *J* = 8.9, 1.5 Hz, 1H), 2.69–2.60 (m, 2H), 2.60–2.53 (m, 2H), 2.51–2.46 (m, 1H), 2.41–2.39 (m, 1H), 2.38–2.34 (m, 1H), 2.09–1.99 (m, 3H), 1.95–1.84 (m, 3H), 1.78–1.68 (m, 3H), 1.56–1.38 (m, 2H), 1.29 (s, H-19, 3H), 1.13 (d, *J* = 7.1 Hz, H-21, 3H), 0.99 (s, H-18, 3H), 0.94 (d, *J* = 6.8 Hz, H-27, 3H), 0.86 (d, *J* = 6.7 Hz, H-26, 3H), 0.84 (d, *J* = 6.8 Hz, H-28, 3H). ^13^C-NMR (101 MHz, MeOD) *δ* 217.53 (q, C-14), 202.21 (q, C-6), 201.68 (q, C-3), 159.33 (q, C-5), 136.00 (CH, C-23), 134.28 (CH, C-22), 126.55 (CH, C-4), 64.05^a^ (q, C-8), 55.68^a^ (q, C-13), 50.96 (CH, C-9), 49.50 (CH, C-17), 44.70 (CH, C-24), 41.42 (CH_2_, C-7), 39.96 (CH_2_, C-1), 38.99 (CH_2_, C-12), 38.63 (CH_2_, C-15), 38.49 (CH, C-20), 37.39 (q, C-10), 35.17 (CH_2_, C-2), 34.37 (CH, C-25), 25.91 (CH_2_, C-11), 24.74 (CH_3_, C-19), 24.18 (CH_3_, C-21), 24.09 (CH_2_, C-16), 20.55 (CH_3_, C-27), 20.14 (CH_3_, C-26), 18.10 (CH_3_, C-28), 17.35 (CH_3_, C-18) (^a^ assignments could be switched).

Compound **2** (8.7 mg) was identified as follows. Demethylincisterol A_3_ (C_21_H_32_O_3_, *m/z* = 331.2271 [M − H]^-^, cald. for 331.2279) [19,20]. ^1^H-NMR (400 MHz, CDCl_3_) *δ* 5.61 (d, *J* = 4.2 Hz, H-2 1H), 5.25 (dd, *J* = 15.2, 7.5 Hz, H-16, 1H), 5.16 (dd, *J* = 15.3, 8.2 Hz, H-15, 1H), 2.68–2.61 (m, H-8, 1H), 2.33–2.26 (m, 1H), 2.06–2.00 (m, 1H), 1.99–1.94 (m, 1H), 1.90–1.80 (m, 3H), 1.71–1.65 (m, 1H), 1.65–1.60 (m, 1H), 1.52–1.42 (m, 4H), 1.03 (d, *J* = 6.2 Hz, H-14, 3H), 0.91 (d, *J* = 6.4 Hz, H-21, 3H), 0.83 (d, *J* = 6.7 Hz, H-19, 3H), 0.82 (d, *J* = 6.6 Hz, H-20, 3H), 0.60 (s, H-12, 3H). ^13^C-NMR (101 MHz, CDCl_3_) *δ* 171.56 (q, C-1), 170.98 (q, C-3), 134.80 (CH, C-15), 132.96 (CH, C-16), 112.29 (CH, C-2), 105.39 (q, C-4), 55.46 (CH, C-11), 50.51 (CH, C-8), 48.98 (q, C-7), 42.96 (CH, C-17), 40.25 (CH, C-13), 35.37 (CH_2_, C-5), 35.19 (CH_2_, C-6), 33.17 (CH, C-18), 28.99 (CH_2_, C-10), 21.50 (CH_2_, C-9), 21.13 (CH_3_, C-14), 20.09 (CH_3_, C-20), 19.78 (CH_3_, C-19), 17.73 (CH_3_, C-21), 11.88 (CH_3_, C-12).

Compound **3** (3.2 mg) was identified as follows. Zinnimidine (C_15_H_19_NO_3_, *m/z* = 262.1435 [M + H]^+^, cald. for 262.1438) [21]. ^1^H-NMR (400 MHz, MeOD) *δ* 7.05 (s, H-6, 1H), 5.49 (t, *J* = 6.6 Hz, H-12, 1H), 4.60 (d, *J* = 6.1 Hz, H-11, 2H), 4.50 (s, H-8, 2H), 3.89 (s, H-9, 3H), 2.16 (s, H-10, 3H), 1.79 (s, H-14, 3H), 1.76 (s, H-15, 3H). ^13^C-NMR (101 MHz, MeOD) *δ* 173.70 (q, C-7), 159.86 (q, C-3), 155.15 (q, C-5), 138.94 (q, C-13), 132.43 (q, C-1), 127.47 (q, C-2), 124.43 (q, C-4), 120.93 (CH, C-12), 101.74 (CH, C-6), 66.59 (CH_2_, C-11), 60.11 (CH_3_, C-9), 45.06 (CH_2_, C-8), 25.87 (CH_3_, C-14), 18.26 (CH_3_, C-15), 9.68 (CH_3_, C-10).

Compound **4** (10.8 mg) was identified as follows. Cyclo-(L-Val-L-Pro) (C_10_H_16_N_2_O_2_, *m/z* = 197.1296 [M + H]^+^, cald. for 197.1285) [22,23,24]. ^1^H-NMR (400 MHz, MeOD) *δ* 4.20 (t, *J* = 7.2 Hz, H-2, 1H), 4.03 (s, H-4, 1H), 3.63–3.45 (m, H-5, 2H), 2.56–2.42 (m, 1H), 2.39–2.20 (m, 1H), 2.07–1.82 (m, 3H), 1.09 (d, *J* = 7.3 Hz, H-9, 3H), 0.93 (d, *J* = 6.9 Hz, H-10, 3H). ^13^C-NMR (101 MHz, MeOD) *δ* 172.60 (q, C-3), 167.57 (q, C-1), 61.51 (CH, C-4), 60.03 (CH, C-2), 46.18 (CH_2_, C-5), 29.87 (CH, C-8), 29.52 (CH_2_, C-7), 23.25 (CH_2_, C-6), 18.85 (CH_3_, C-9), 16.65 (CH_3_, C-10).

Compound **5** (7.4 mg) was identified as follows. Cyclo-(L-Ile-L-Pro) (C_11_H_18_N_2_O_2_, *m/z* = 211.1413 [M + H]^+^, cald. For 211.1441) [25,26]. ^1^H-NMR (400 MHz, MeOD) *δ* 4.19 (t, *J* = 7.0 Hz, H-2, 1H), 4.07 (s, H-4, 1H), 3.60–3.45 (m, H-5, 2H), 2.36–2.26 (m, H-8, 1H), 2.20–2.12 (m, H-7a, 1H), 2.05–1.98 (m, H-7b, 1H), 1.97–1.87 (m, H-6, 2H), 1.50–1.27 (m, H-9, 2H), 1.06 (d, *J* = 7.2 Hz, H-10, 3H), 0.93 (t, *J* = 7.4 Hz, H-11, 3H). ^13^C-NMR (101 MHz, MeOD) *δ* 172.45 (q, C-3), 167.59 (q, C-1), 61.30 (CH, C-4), 59.99 (CH, C-2), 46.17 (CH_2_, C-5), 37.06 (CH, C-8), 29.54 (CH_2_, C-7), 25.41 (CH_2_, C-9), 23.22 (CH_2_, C-6), 15.52 (CH_3_, C-10), 12.57 (CH_3_, C-11).

Compound **6** (7.5 mg) was identified as follows. Cyclo-(L-Leu-L-Pro) (C_11_H_18_N_2_O_2_, *m/z* = 211.1409 [M + H]^+^, cald. for 211.1441) [26,27]. ^1^H-NMR (400 MHz, MeOD) *δ* 4.25 (t, *J* = 7.1 Hz, H-2, 1H), 4.12 (dd, *J* = 6.8, 3.7 Hz, H-4, 1H), 3.56–3.44 (m, H-7, 2H), 2.36–2.23 (m, 1H), 2.10–1.78 (m, 5H), 1.58–1.44 (m, 1H), 0.95 (dd, *J* = 6.3, 2.5 Hz, H-10, 11, 6H). ^13^C-NMR (101 MHz, MeOD) *δ* 172.81 (q, C-3), 168.92 (q, C-1), 60.28 (CH, C-4), 54.63 (CH, C-2), 46.44 (CH_2_, C-5), 39.39 (CH_2_, C-8), 29.07 (CH_2_, C-7), 25.76 (CH, C-9), 23.65 (CH_2_, C-6), 23.30 (CH_3_, C-10), 22.20 (CH_3_, C-11).

### 2.3. The Growth Profiles of B. cereus at Sub-Minimum Inhibitory Concentrations of Demethylincisterol A_3_

**Figure 3:**
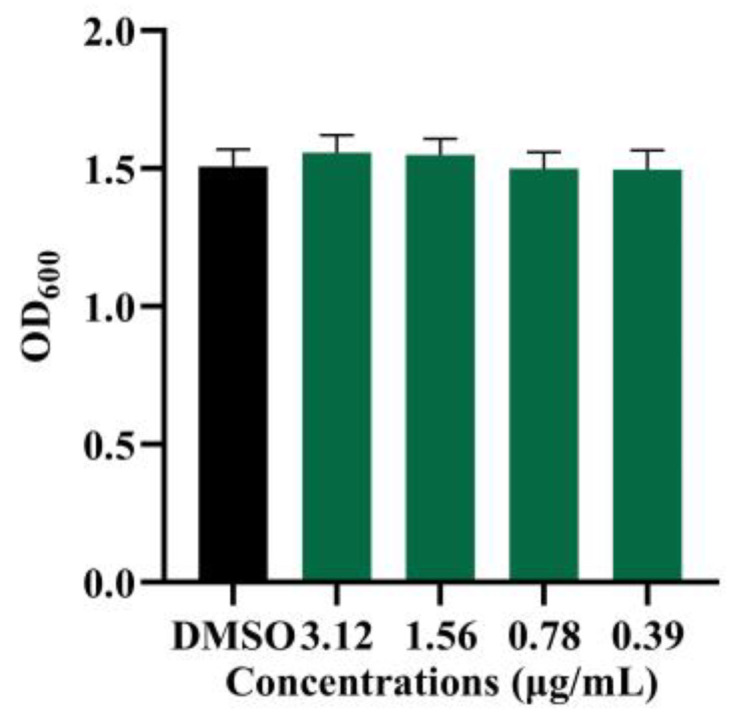
The growth of *B. cereus* at sub-MICs of demethylincisterol A_3_. *B. cereus* grew for 24 h in different sub-MICs (3.12, 1.56, and 0.78 μg/mL) of demethylincisterol A_3_; DMSO was used as the negative control. Error bars represent the standard deviation (*n* = 3).

After quorum sensing inhibitory (QSI)-screening the isolated compounds with *B. cereus*, it was found that only demethylincisterol A_3_ had antibacterial and QSI activities. Its minimum inhibitory concentration (MIC) was 6.25 μg/mL. At sub-MICs (3.12, 1.56, and 0.78 μg/mL), there was no effect on the growth of *B. cereus* (Figure 3).

### 2.4. Demethylincisterol A_3_ Inhibits the Biofilm Formation of B. cereus

**Figure 4:**
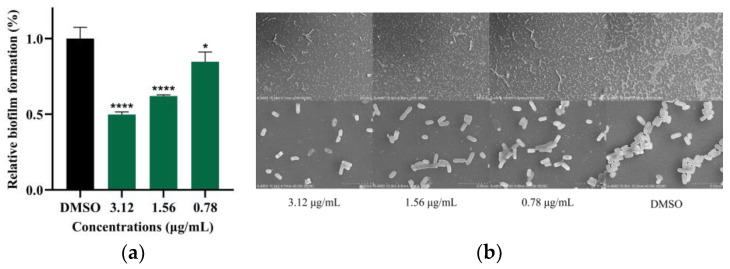
The effect of demethylincisterol A_3_ on the biofilm formation of *B. cereus*. *B. cereus* was treated with different concentrations (3.12, 1.56, and 0.78 μg/mL) of demethylsterol A_3_ for 24 h, with DMSO as the negative control. (**a**) Biofilm formation. (**b**) SEM images of biofilms. Error bars represent the standard deviation (*n* = 3). * *p* < 0.05, **** *p* < 0.0001.

The results of crystal violet staining indicated a significant reduction in biofilm formation in the demethylincisterol A_3_ treatment group when compared to the control group (Figure 4a). When the demethylincisterol A_3_ concentration was 3.12, 1.56, and 0.78 μg/mL, the biofilm decreased by 50.2%, 37.9%, and 15.2%, respectively. A substantial reduction in bacterial density after treatment with demethylincisterol A_3_ was observed using scanning electron microscopy (SEM) (Figure 4b).

### 2.5. Demethylincisterol A_3_ Inhibits the Swimming of B. cereus

**Figure 5:**
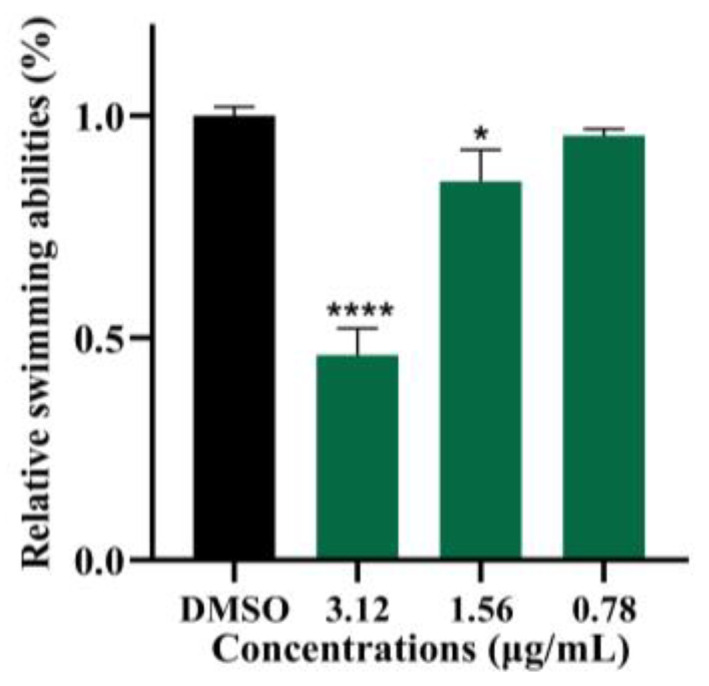
The effect of demethylincisterol A_3_ on the swimming of *B. cereus* was examined using a swimming medium. The swimming behavior of *B. cereus* for 24 h of treatment with demethylsterol A_3_ at various concentrations (3.12, 1.56, and 0.78 μg/mL) was tested. DMSO served as a negative control. After 24 h of incubation, the swimming diameter was observed and recorded. Values are represented as mean ± standard deviation (*n* = 3). Significant differences are denoted by * *p* < 0.05 and **** *p* < 0.0001, respectively.

The swimming behavior of *B. cereus* was notably hindered after exposure to demethylincisterol A_3_ (Figure 5). Under the action of demethylincisterol A_3_ at 3.12, 1.56, and 0.78 μg/mL, the swimming ability of *B. cereus* was reduced by 53.9%, 14.7%, and 4.4%, respectively.

### 2.6. Demethylincisterol A_3_ Inhibits the Protease and Hemolytic Activity of B. cereus

**Figure 6:**
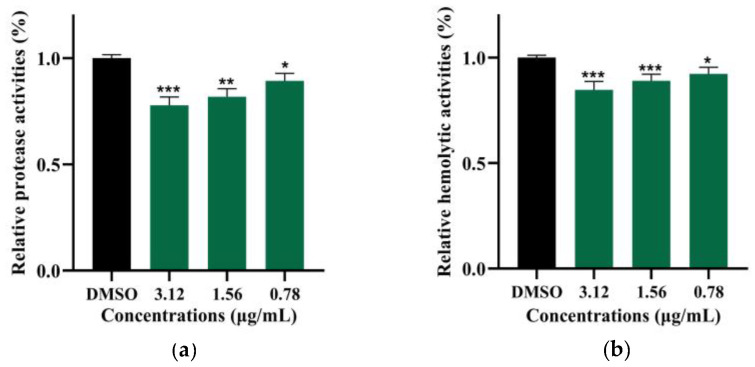
The effect of demethylincisterol A_3_ on the virulence factors of *B. cereus*. The virulence factor produced by *B. cereus* after 24 h of treatment with different concentrations (3.12, 1.56, and 0.78 μg/mL) of demethylsterol A_3_ was evaluated. DMSO was used as a negative control. (**a**) The protease activity was assessed by examining the clear zone on the skim milk agar plates. (**b**) The hemolysin activities were analyzed by examining the hemolysis zone on the blood agar plates. Values are represented as mean ± standard deviation (*n* = 3). Significant differences are denoted by * *p* < 0.05, ** *p* < 0.01, and *** *p* < 0.001, respectively.

After treatment with demethylincisterol A_3_, the protease of *B. cereus* was significantly inhibited (Figure 6a). Under the action of demethylincisterol A_3_ at 3.12, 1.56, and 0.78 μg/mL, the protease activities of *B. cereus* were reduced by 22.1%, 18.1%, and 10.7%, respectively. The hemolytic activity of *B. cereus* was significantly inhibited when treated with demethylincisterol A_3_ (Figure 6b). Under the action of demethylincisterol A_3_ at 3.12, 1.56, and 0.78 μg/mL, the hemolytic activities of *B. cereus* were reduced by 15.3%, 11.0%, and 7.6%, respectively. 

### 2.7. Demethylincisterol A_3_ Inhibits the Expression of Key Virulence-Related Genes of B. cereus

**Figure 7:**
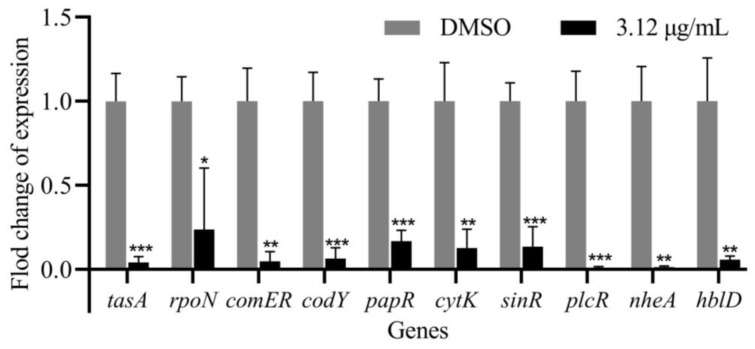
The effect of demethylincisterol A_3_ on *B. cereus* gene transcription was investigated. The expression of QS-related genes in *B. cereus* was examined after treatment with 3.12 μg/mL of demethylsterol A_3_ for 24 h. DMSO was the negative control. Three biological replicates were conducted and are represented as mean ± standard deviation (*n* = 3). Significant differences are denoted by * *p* < 0.05, ** *p* < 0.01, and *** *p* < 0.001, respectively.

The expression of virulence-related genes of *B. cereus* was significantly inhibited when treated with demethylincisterol A_3_ (Figure 7). Under the action of demethylincisterol A_3_ at 3.12 μg/mL, the expression of the *sinR*, *tasA*, *papR*, *cytK*, *rpoN*, *hblD*, *comER*, *codY*, *plcR*, and *nheA* genes in *B. cereus* was significantly reduced.

### 2.8. Demethylincisterol A_3_ Reduces the Mortality Rate of B. cereus

**Figure 8:**
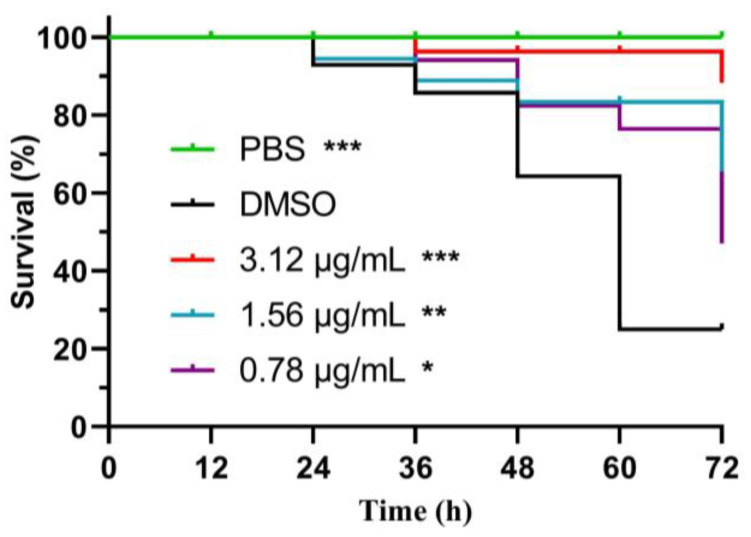
The effect of demethylincisterol A_3_ on the survival rate of *G. mellonella* larval models was observed. PBS was the blank control. DMSO was used as a negative control. Statistical analyses were carried out using the log-rank (Mantel–Cox) test. Three biological replicates were conducted and are represented as mean ± standard deviation (*n* = 3). Significant differences are denoted by * *p* < 0.05, ** *p* < 0.01, and *** *p* < 0.001, respectively.

As shown in Figure 8, the supernatant of *B. cereus* treated with demethylincisterol A_3_ was injected into a *Galleria mellonella* larval model. Only 25% of the larvae in the DMSO group survived after 72 h. However, the survival rate of the experimental group treated with demethylincisterol A_3_ was significantly improved, with a survival rate of 88% for the larvae at a concentration of 3.12 μg/mL.

## 3. Discussion

In this study, demethylincisterol A_3_, along with five other compounds, was initially isolated from *P. sacchari*. As an incisterol-type steroid, demethylincisterol A_3_ has been identified in various fungi, possibly representing a common metabolite in fungal processes [28,29,30]. Notably, demethylincisterol A_3_ exhibits significant anti-tumor and anti-inflammatory effects [31,32]. Studies have demonstrated that the rate of inhibition exhibited by demethylincisterol A_3_ towards *Helicobacter pylori* is 11% greater than that of 100 μM quercetin [33]. Despite its recognized therapeutic potential, there has been limited research on its QS inhibitory effect against pathogens. Therefore, this study pioneers the investigation of demethylincisterol A_3_’s impact on QS, biofilm formation, and virulence factors in *B. cereus*.

At the MIC, *B. cereus* growth was inhibited, but at sub-MICs, there was no significant effect (Figure 3). *B. cereus* biofilms contribute to equipment adhesion, increased resistance, and toxicity [34]. Following demethylincisterol A_3_ treatment, biofilm formation was significantly inhibited, decreasing production by 50.2% at 3.12 μg/mL. Our research is consistent with that pertaining to Siphonocholin, which can effectively inhibit biofilm formation at 1/2 MIC [35]. At the same time, diallyl disulfide can also inhibit the biofilm formation of *B. cereus* at 1/2 MIC [36]. The downregulation of *rpoN* and *comER*, important regulators of *B. cereus* biofilms, is associated with reduced biofilm production [37,38]. Additionally, the decrease in *tasA* expression directly correlates with a reduction in biofilm matrix protein synthesis [39].

*B. cereus* utilizes its swimming ability to navigate and reach the target host [36], and this mobility is intertwined with its biofilm formation [16]. Upon the addition of demethylincisterol A_3_, both the swimming ability and the biofilm production of *B. cereus* decreased. Genes associated with flagella or mobility, including *rpoN*, *codY*, and *sinR* [38,40], showed significant downregulation after demethylincisterol A_3_ treatment. 

Proteases and hemolysin serve as virulence factors produced by *B. cereus*, aiding in immune system evasion [41]. Hemolysins induce red blood cell rupture, leading to hemolysis [42]. Following demethylincisterol A_3_ treatment, *B. cereus* exhibited a significant reduction in protease and hemolysin production. In *B. cereus*, *nhe* represents non-hemolytic enterotoxin, *hbl* represents hemolysin BL, and *cytK* represents cytotoxin, regulated by *plcR* [42]. *papR* can produce PapR peptides to activate the PlcR protein [43]. Demethylincisterol A_3_ reduced the mortality rate of *B. cereus* in the *G. mellonella* larval model from 75% to 12%. Furthermore, demethylincisterol A_3_ diminished the transcription of the *nheA*, *hblD*, *cytK*, *plcR*, and *papR* genes in *B. cereus*. Therefore, demethylincisterol A_3_ may mitigate the production of virulence factors in *B. cereus*, weakening its pathogenicity by inhibiting the transcription of the *nheA*, *hblD*, *cytK*, *plcR*, and *papR* genes.

In the current study, cyclo-(L-Pro-L-Leu) was also extracted from *P. sacchari*. This cyclodipeptide was identified as a signaling molecule that facilitates communication between *B. cereus* and *Cronobacter sakazakii* [44]. It was already known whether or not it was a molecular signaling pathway in *B. cereus*; further research will be conducted to determine the above.

## 4. Materials and Methods

### 4.1. General

*B. cereus* (ATCC11778) was cultured using LB medium at 37 °C. Fresh *Laurencia* sp. was collected from the Nansha Islands area in the South China Sea (Figure S1), identified by Prof. X.L. Wang (Institute of Oceanology, CAS, China). The test compound was firstly dissolved in dimethyl sulfoxide (DMSO) to 1 mg/mL and sterilized using a filter membrane (0.22 μm) to obtain the stock solution. During all the experiments, an equal volume of DMSO was added as the negative control. The NMR was the Bruker Avance neo 400 (Bruker BioSpin AG, Ettlingen, Germany). The MS was ion trap time-of-flight mass spectrometry liquid chromatography-mass spectrometry (Shimadzu, Kyoto, Japan). All used chemicals in this work were analytical reagents.

### 4.2. Seaweed Sample Collection and Morphological Identification

The seaweed samples were collected by researcher Zhi-Kai Guo from the Institute of Biology, Chinese Academy of Tropical Agricultural Sciences, from the reef of Zhaoshu Island in the Xisha Xuande Islands. The algae species were identified by Professor Xu-Lei Wang from the Qingdao Institute of Oceanography, Chinese Academy of Sciences. The algal body was purplish red in appearance, with cylindrical branches—mostly forked or trifurcate branches, dense and clustered—with forked or threefold apical branchlets, and a concave apical center with trichonema. The tetrasporangium divides cruciform or conical. Identified as *Laurencia* sp. (Figure S1). Endophytes were extracted from algae. After placing fresh *Laurencia* sp. in a petri dish and disinfecting it three times with sterile water, it was rinsed with sterile water and alcohol for 30 seconds. It was then immersed in a 1% (*w*/*w*) NaClO solution for three minutes and then rinsed five times with sterile water. Once it was dried with filter paper, it was chopped into small segments. Mixed the small segments with sterile quartz sand. Then grinded the mixture in a sterile phosphate-buffered saline (PBS) environment. Diluted the grinding solution using a PBS gradient. A layer of 100 μL dilution solution (evenly distributed) was coated onto the culture plate containing PDA, Gause’s Synthetic Agar No.1 (GSA) and LB. LB plate was cultured at 37 °C, while PDA and GSA plates were cultured at 28 °C. Colonies with different morphologies were selected for purification and preservation during the cultivation process.

### 4.3. Identification of QSI Active-Strain W2-F1

The QSI activities of the crude extract of ethyl acetate from the isolated strain were evaluated by using the indicator strain *Chromobacterium violaceum* CV026. Among these 7 isolates, W2-F1 showed the best QSI activity (Figure S2). The morphological characteristics of strain W2-F1 cultivated in PDA medium (Hope Bio, Qingdao, China) were examined [45]. Further, the genomic DNA was extracted and amplified using ITS primers (5′-TCCGTAGGTGGAACCTGGG-3′ and 5′-TCTCCGCTTATTGCT-3′) and the obtained sequence was aligned in the NCBI. Based on the sequence similarities, the phylogenetic tree was constructed by the neighbor-joining method with the 1000-bootstrap value using MEGA 7.0 software [46]. The W2-F1 strain was identified as *P. sacchari*.

### 4.4. Scale-Up Fermentation of QSI Active-Strain P. sacchari and Isolation, Purification, and Elucidation of Compounds under Bio-Guided Screenings

*P. sacchari* was activated on a PDA plate, then inoculated into a solid PDA medium containing 3% sea salt and cultured at 28 °C for 14 days. After the culture was completed, the mycelium was broken together with the culture medium. Absolute ethanol was added to the mixture and treated with ultrasonic for 12 h and then filtered, repeated three times. The ethanol in the filtrate was removed by evaporation under reduced pressure. The residual liquid was extracted three times by equal ethyl acetate (EtOAc) and concentrated under reduced pressure to give a crude extract (110.1 g), which was subjected to column chromatography (CC) over a silica gel (100–200 mesh) eluted with a gradient of CH_2_Cl_2_/MeOH (100:0–0:100) to obtain fractions (R1-R11). The fraction R4 (5.26 g) showing QSI activity was further submitted to silica gel (200–300 mesh) column chromatography (CC) and eluted with a gradient system of petroleum ether/CH_2_Cl_2_ (20:1–1:1) and CH_2_Cl_2_/MeOH (100:1–10:1) to obtain twenty-two subfractions (R4–1–22). Fraction R4–9 (0.24 g) showing QSI activity was purified by reversed-phase C_18_ CC and semi-preparation HPLC equipped with a preparative column (5 μm, 21.2 × 250mm; Beijing, China) to obtain compounds **1** and **2** (eluting conditions: 100% MeOH, flow rate = 8 mL/min; yields: 4.8 and 8.7 mg). QSI-active R4–18 were eluted by silica gel column chromatography and semi-preparation HPLC to obtain compound **3** (eluting conditions: 90% MeOH, flow rate = 8 mL/min; yield: 3.2 mg) and R4–18–6. Compound **4** (yield: 10.8 mg) was obtained from R4–18–6 with a 60% MeOH eluting solution and a flow rate of 8 mL/min. Compounds **5** and **6** (yields: 7.4 and 7.5 mg) were obtained from R4–18–6 with a 50% MeOH elution solution and flow rate of 6 mL/min.

### 4.5. MIC of Demethylincisterol A_3_ against B. cereus

MIC of demethylincisterol A_3_ was measured by using the double dilution method [35]. The seed solution of *B. cereus* that was cultured overnight was added to LB medium at a rate of 1% (*v*/*v*). The compounds were added and the mixture was diluted and incubated at 37 °C and 180 rpm for 24 h. DMSO was used as the negative control. Growth profiles were measured at 620 nm.

### 4.6. Growth Measurement of B. cereus

The planktonic cell growth measurement was performed by the reported methods, with some modifications [47]. The *B. cereus* culture was inoculated into LB medium at a ratio of 1% (*v*/*v*). Demethylincisterol A_3_ was then added to achieve final concentrations of 3.12, 1.56, and 0.78 μg/mL. After 24 hours of cultivation at 37 °C and 180 rpm, OD_620_ was measured (Biotech Epoch 2, Santa Clara, CA, USA).

### 4.7. The Effects of Biofilm Formation

The biofilm formation assays were conducted following established procedures with slight modifications, as outlined in the report by [48]. *B. cereus* cultures that had been grown overnight were introduced into LB medium at a 1% (*v*/*v*) ratio, and demethylincisterol A_3_ was added. A 200 µL aliquot of this mixed culture was then transferred to a 96-well polystyrene microtiter plate. The final concentrations of demethylincisterol A_3_ in the wells were 3.12, 1.56, and 0.78 μg/mL. The plate was then incubated at 37 °C for 24 h. After this period, the culture medium was carefully removed and the wells were washed three times with sterile PBS to eliminate planktonic cells. The remaining water in the wells was evaporated at 60 °C. The biofilms that had adhered to the bottom of the wells were fixed with cool methanol for 15 min. The liquid was then removed and the plate was dried again at 60 °C. Each well was stained with 200 µL of a 0.05% (*w*/*v*) crystal violet solution. After 10 min staining, any excess crystal violet was removed and the wells were washed three times with PBS. Once the plate had dried at 60 °C, 200 µL of 95% (*v*/*v*) ethanol was added to each well and the plate was decolorized for 15 min on a shaker set at 37 °C and 180 rpm. A 150 µL sample of the decolorizing solution was then taken from each well and the absorbance was measured at 570 nm.

SEM measurement: The coating was fixed on the glass slide with 2.5% (*v*/*v*) glutaraldehyde for 12 h. After fixation, it was washed at once with ultrapure water and then gradient dehydration was performed with 50%, 60%, 70%, 80%, 90%, and 100% (*v*/*v*) ethanol in sequence. After drying, it was sprayed gold and observed under a SEMn microscope.

### 4.8. Swimming Motility Assay

The swimming motility assay was evaluated by established protocols and reports, as per the study by [46]. Swimming medium (10 g/L peptone, 5 g/L NaCl, 3 g/L agar) was added to demethylincisterol A_3_ to the final concentrations of 3.12, 1.56, and 0.78 μg/mL, respectively. It was mixed well and poured evenly into a petri dish. Sterile water served as a negative control. After solidification, 2 μL of *B. cereus* bacterial solution was inoculated in the center of the swimming medium. The migration diameter was recorded after 24 h of cultivation at 37 °C.

### 4.9. Measurement of Protease Production

Protease activities were measured by reported methods, with some modifications [49]. *B. cereus* cultures that had been grown overnight were introduced into LB medium at a 1% (*v*/*v*) ratio, and demethylincisterol A_3_ was added. The final concentrations of desmethylergosterol A_3_ were 3.12, 1.56, and 0.78 μg/mL, respectively. The solution was incubated at 37 °C and 180 rpm for 24 h. The fermentation broth was centrifuged (8000× *g*, 4 °C, 15 min) to collect the supernatant. The supernatant was filtered through a 0.22 μm membrane. Skim milk agar was prepared (1/4-strength LB broth with 4% (*w*/*v*) skim milk and 1.5% (*w*/*v*) agar). Added 50 μL of supernatant to the wells of a skim milk agar plate and incubated at 37 °C for 24 h. The protease activity was assessed by examining the clear zone on the skim milk agar plates.

### 4.10. Measurement of Hemolytic Activities

Hemolytic activities were measured by reported methods, with some modifications [50]. Hemolysis experiments were performed using a trypticase soy sheep blood agar plate (Huankai Microbial, Guangzhou, China). The supernatant was prepared according to the previous method. Then, 50 µL of cultured supernatant was added after drilling, incubated at 37 °C for 24 h, and the size of the hemolytic halo was observed.

### 4.11. Quantitative Real-Time PCR

Quantitative real-time PCR (RT-qPCR) was performed according to reported methods, with minor modifications [48]. The bacterial solution cultured overnight was diluted to a 0.5 Michaelis turbidimetric unit with sterile LB broth. Overnight cultured *B. cereus* was added in a 1% (*v*/*v*) ratio to LB medium. The experimental group was supplemented with demethylincisterol A_3_ to make its concentration 3.12 μg/mL, and the same volume of DMSO was used as the negative control group. The solution was incubated at 37 °C and 180 rpm for 24 h. The fermentation broth was centrifuged (8000× *g*, 4 °C, 15 min) to collect cells and washed thrice with diethylpyrocarbonate (DEPC)-treated water. The total RNA of *B. cereus* was extracted using an Eastern^®^ Super Total RNA Extraction Kit (Promega, Beijing, China) and transformed into cDNA by a Reverse Transcription Kit (Biosharp, Beijing, China). The real-time PCR reaction was performed using a TB Green^®^ Premix Ex Taq™ II FAST qPCR Mix (TaKaRa, Dalian, China) with a Bio-Rad CFX Connect System (Bio-Rad, Hercules, CA, USA). The primers used for real-time PCR are listed in Table 1. Gene *16S* rRNA was used as an internal control [36].

### 4.12. Evaluating the Inhibitory Effect of Demethylincisterol A_3_ in a G. mellonella Model

A *G. mellonella* larval model was used to evaluate the toxic inhibitory effect of demethylincisterol A_3_ on *B. cereus* [51]. The supernatant was prepared as described above. The larvae were randomly divided into 15 groups (*n* = 10 per group) and 5 μL of supernatant was injected into the posterior part of the left abdominal foot. Then, the larvae were cultured at room temperature for 72 h, and their growth was recorded every 12 h. PBS served as a blank control.

### 4.13. Statistical Analysis

Unless otherwise stated, all experiments described were performed in triplicate independently. The data shown in the figures are the mean ± standard deviation (± SD) of the replicates. Statistical differences were determined using one-way ANOVA or *t*-test using GraphPad Prism 8, where *p* < 0.05 was considered statistically significant.

## 5. Conclusions

Demethylincisterol A_3_ exhibits significant inhibitory effects on *B. cereus*, encompassing reduced biofilm formation, diminished bacterial mobility, and lowered production of virulence factors. This multifaceted impact has the potential to attenuate the pathogenicity of *B. cereus*. Based on these results, it can be inferred that demethylincisterol A_3_ exhibits potential as a valuable compound in the development of novel antibacterial therapies.

## Figures and Tables

**Table 1:** PCR primers for RT-qPCR.

Primer	Sequence (5′-3′)
*16S* rRNA_F	GGAGGAAGGTGGGGATGAC
*16S* rRNA_R	ATGGTGTGACGGGCGGTGTG
*sinR*_F	AGCGAGCGCCGATATGATAG
*sinR*_R	TCGAGCGCATTCGTAACCAT
*tasA*_F	ACTCGCCACATGGAAACACA
*tasA*_R	ACGATTTGTTCGTTTTCTTCGT
*papR*_F	TGTACCTCTTGATCACTGTGAGA
*papR*_R	AACGTTAGCAATGGCATGGG
*cytK*_F	CGATGACCCAAGCGCTGATA
*ctyK*_R	GTTGCACTAGCACCAGGGAT
*rpoN*_F	CACTTGAACGAGCTTTCGCC
*rpoN*_R	GGGGCGCGTAATATTCAGGA
*hblD*_F	GGTCCAGATGGGAAAGGTGG
*hblD*_R	AAGTTGTGGGATCGTTGCCT
*comER*_F	CAAGTTGCGGTCCTGCTTTC
*comER*_R	AATTTCCCCATCCCCACGAC
*codY*_F	CCACGACGGCTAACTACGAA
*codY*_R	GCGTTATTACAGAGCGCAGC
*plcR*_F	GGGTGATGCGGGGATTAACA
*plcR*_R	GGCTCACTTCCGATTGGTGA
*nheA*_F	TCTTGCAACAGCCAGACATT
*nheA*_R	CTCTCGCACATTCGCCTTTG

## Data Availability

The data presented in this study are available from the corresponding author upon request.

## Supplementary Materials

The following supporting information can be downloaded at https://www.mdpi.com/article/10.3390/md22040161/s1: Figure S1: *Laurencia* sp. was collected from the Nansha Islands area in the South China Sea; Figure S2: Evaluated QSI activities using the indicator strain of *Chromobacterium violaceum* CV026; Figure S3–S8: ^1^H, ^13^C NMR, and HRMS data of compounds **1**–**6**; Figure S9: Sequence comparison results of strain W2-F1; Figure S10–S13: Preparation spectrum of compounds **1**–**6**; Table S1: The chemical shifts of each hydrogen-bearing chiral carbon.
