# Supplementary material for: An Investigation of Quorum Sensing Inhibitors against Bacillus cereus in The Endophytic Fungus Pithomyces sacchari of the Laurencia sp
Source: Mar Drugs. 2024 Mar 31;22(4):161. doi: 10.3390/md22040161 (PMC11051030; doi:10.3390/md22040161)
Supplement: Supplementary file 1 [file marinedrugs-22-00161-s001.zip › marinedrugs-2905342-supplementary.pdf]

SUPPLYMENTARY DATA

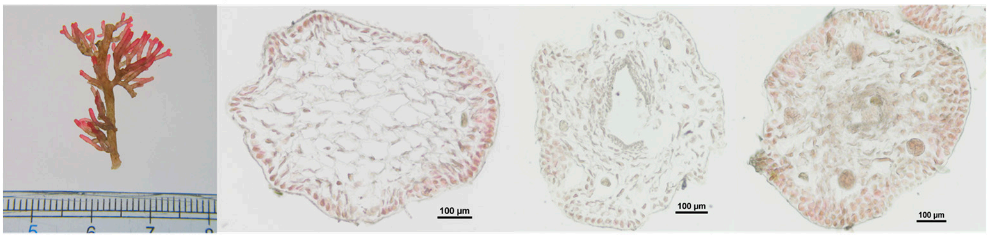

Figure S1. *Laurencia* sp. was collected from the Nansha Islands area in the South China Sea.

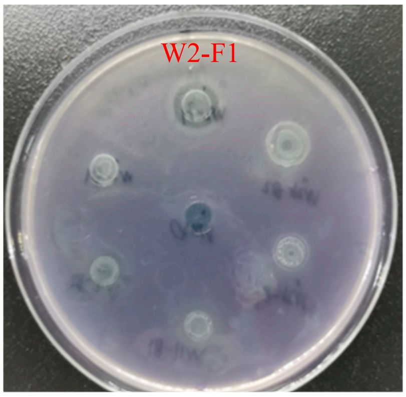

Figure S2. Evaluated QSI activities using the indicator strain of *Chromobacterium violaceum* CV026.

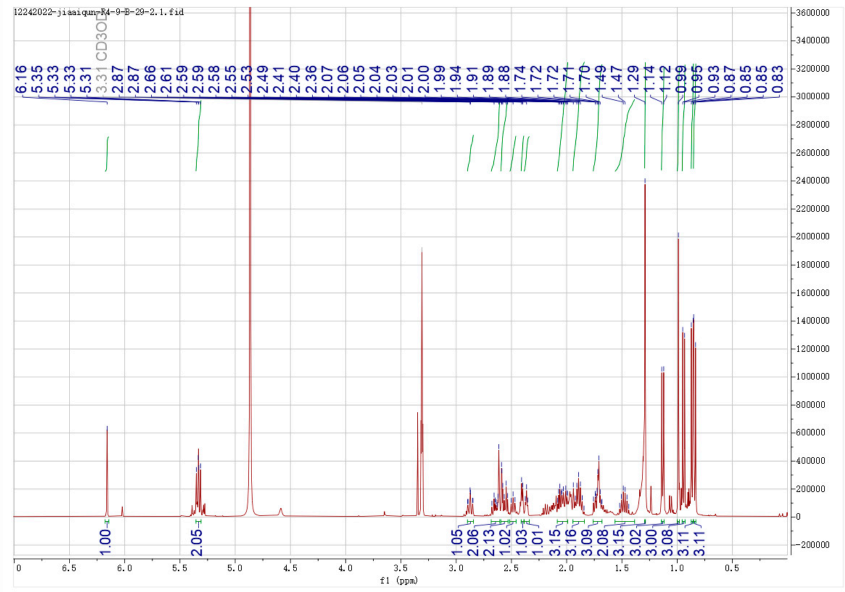

Figure S3-1. ^{1}H NMR spectrum (400 MHz) of compound **1** in MeOD.

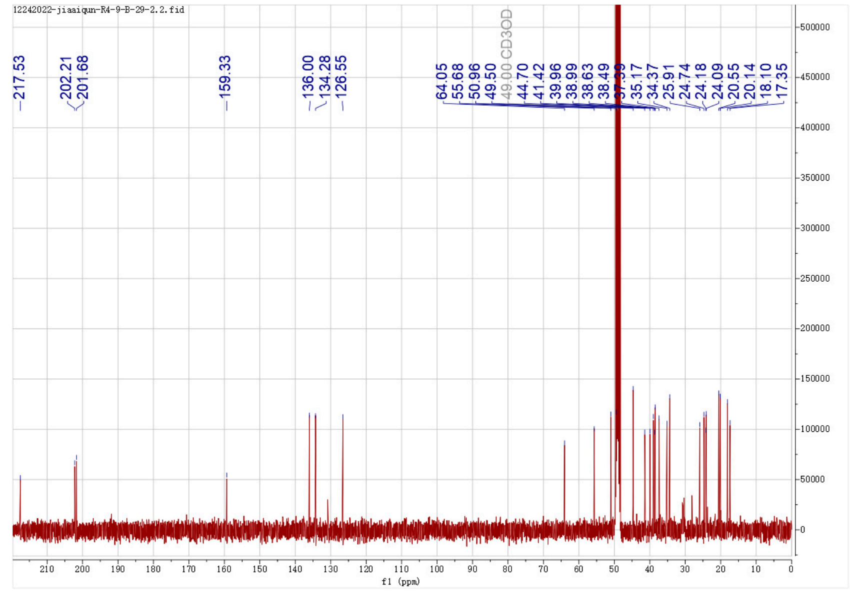

Figure S3-2. ^{13}C NMR spectrum (101 MHz) of compound **1** in MeOD.

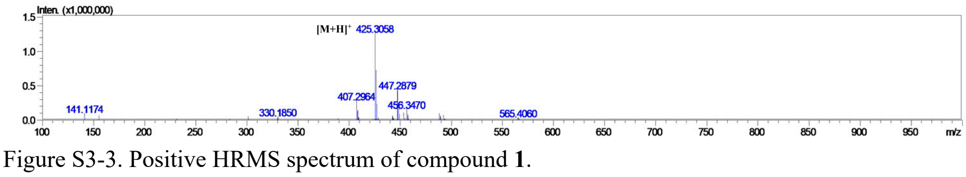

Figure S3-3. Positive HRMS spectrum of compound **1**.

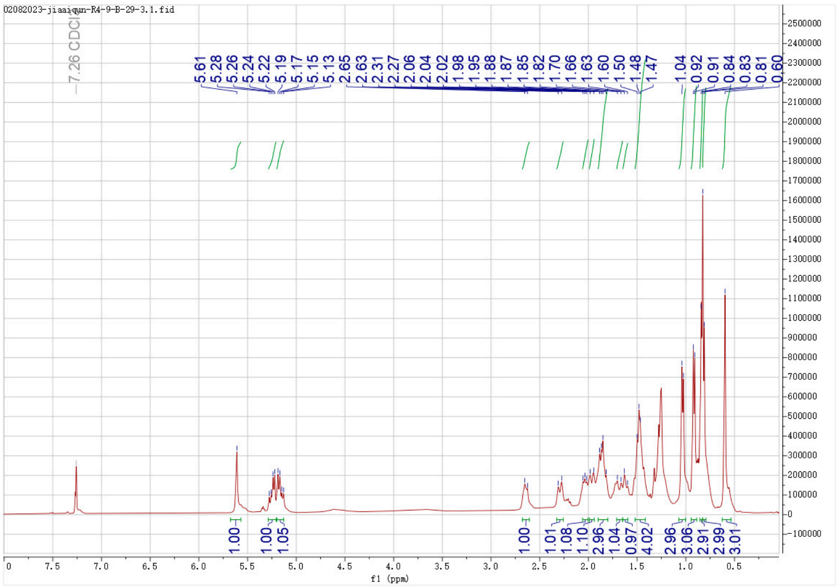

Figure S4-1. ^{1}H NMR spectrum (400 MHz) of compound **2** in $CDCl_3$.

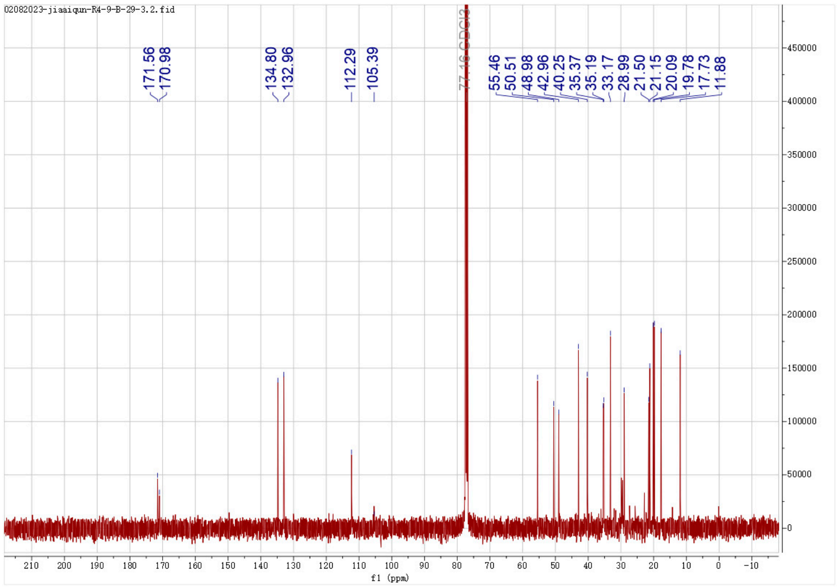

Figure S4-2. ^{13}C NMR spectrum (101 MHz) of compound **2** in $CDCl_3$.

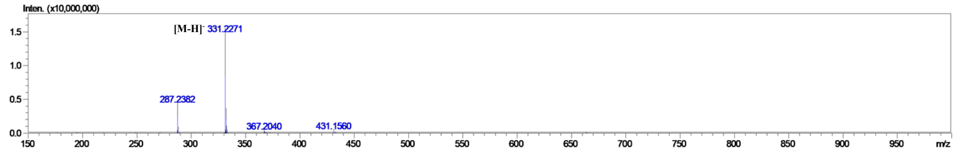

Figure S4-3. Negative HRMS spectrum of compound **2**.

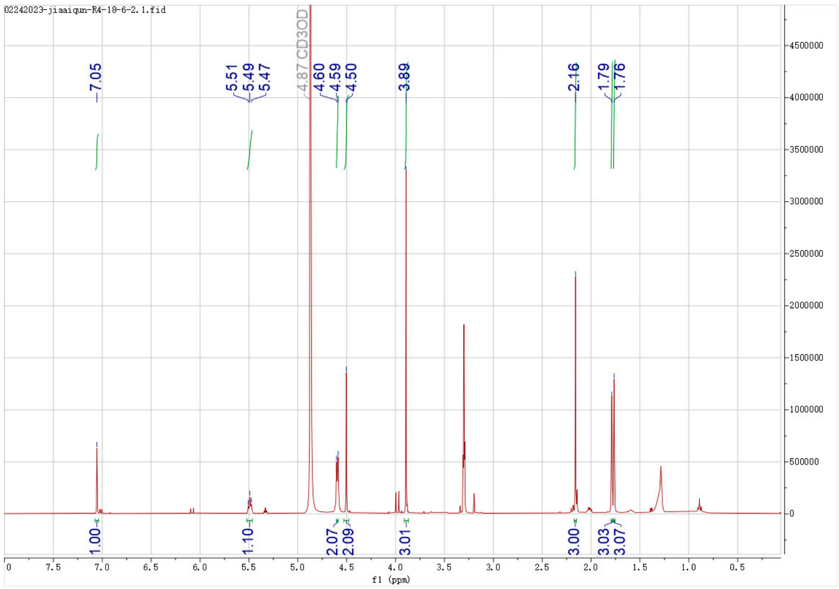

Figure S5-1. ^{1}H NMR spectrum (400 MHz) of compound **3** in MeOD.

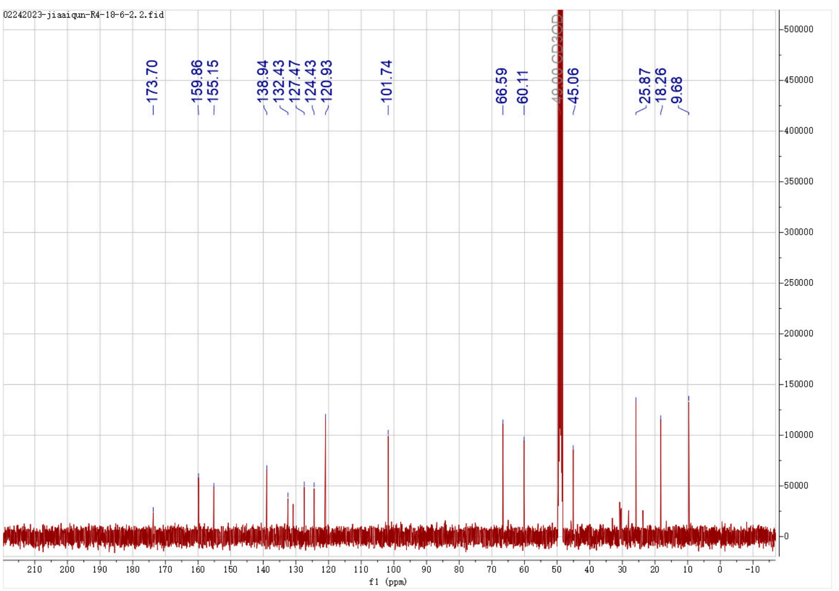

Figure S5-2. ^{13}C NMR spectrum (101 MHz) of compound **3** in MeOD.

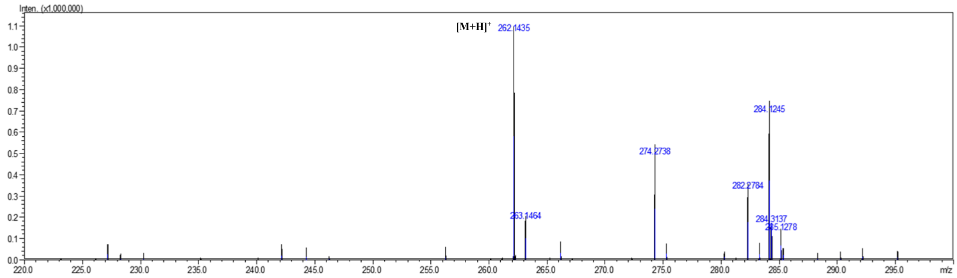

FigureS5-3. Positive HRMS spectrum of compound **3**.

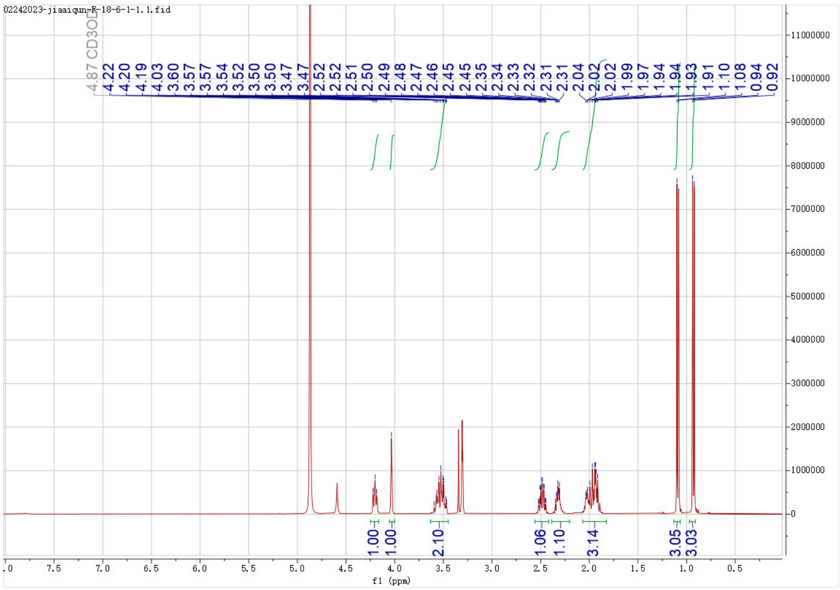

Figure S6-1. ^{1}H NMR spectrum (400 MHz) of compound **4** in MeOD.

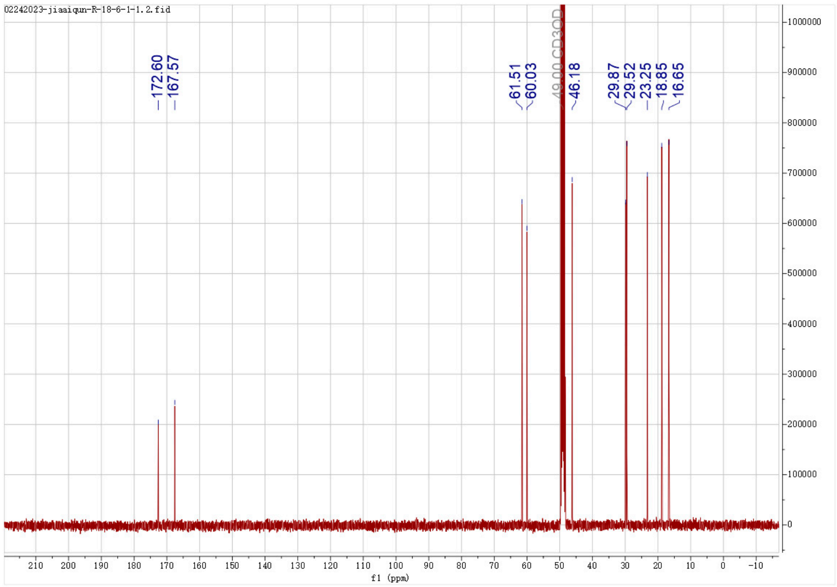

Figure S6-2. ^{13}C NMR spectrum (101 MHz) of compound **4** in MeOD.

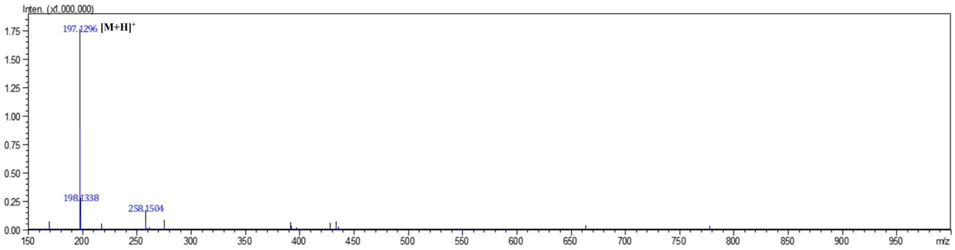

Figure S6-3. Positive HRMS spectrum of compound **4**.

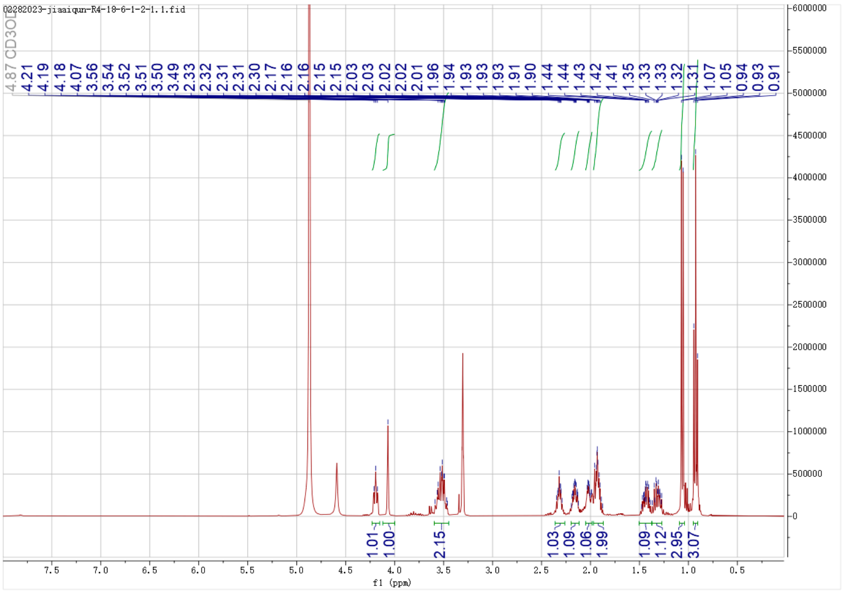

Figure S7-1. ^{1}H NMR spectrum (400 MHz) of compound **5** in MeOD.

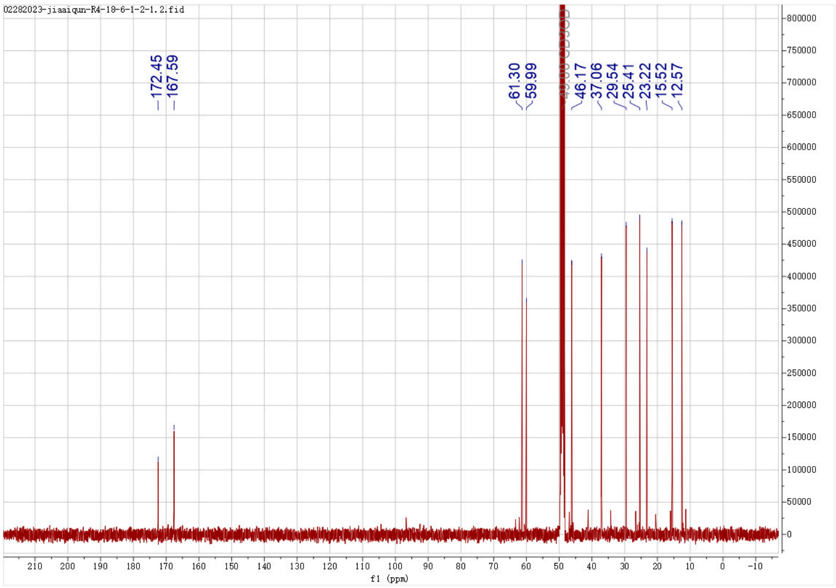

Figure S7-2. ^{13}C NMR spectrum (101 MHz) of compound **5** in MeOD.

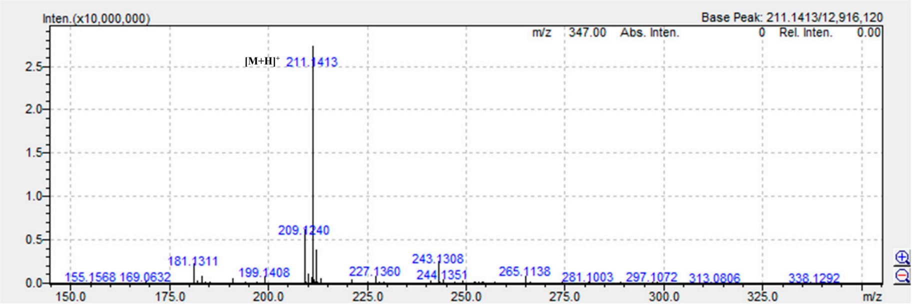

Figure S7-3. Positive HRMS spectrum of compound **5**.

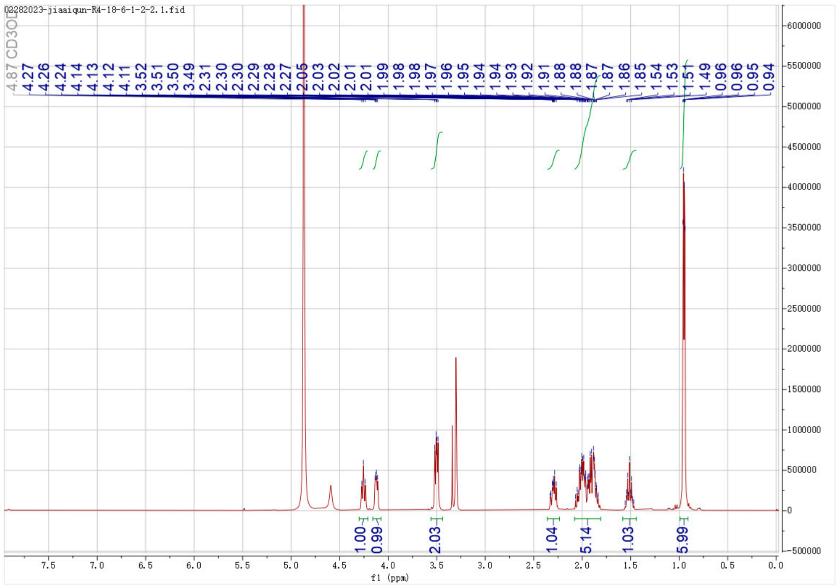

Figure S8-1. ^{1}H NMR spectrum (400 MHz) of compound **6** in MeOD.

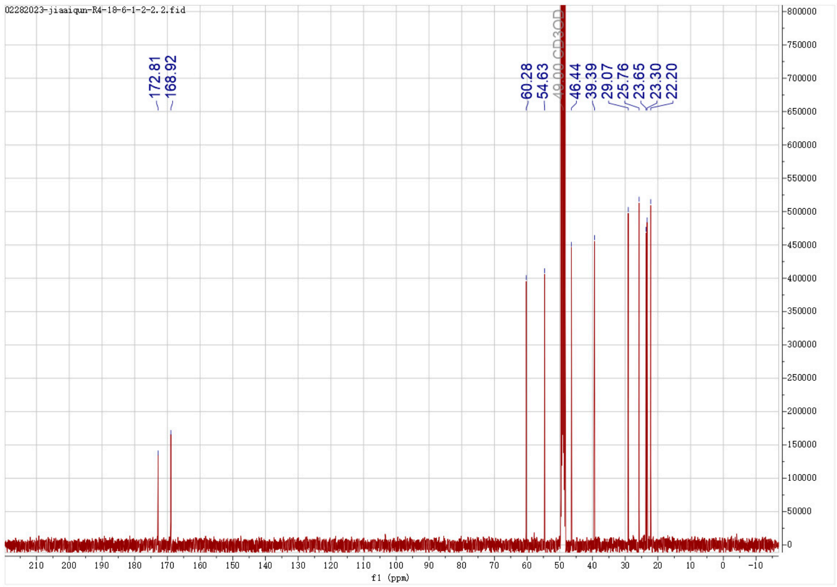

Figure S8-2. ^{13}C NMR spectrum (101 MHz) of compound **6** in MeOD.

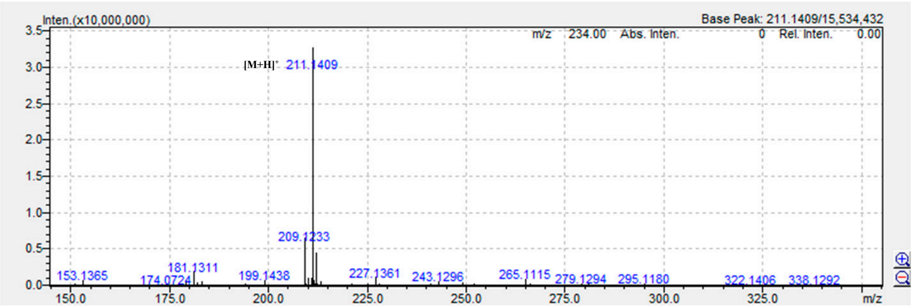

Figure S8-3. Positive HRMS spectrum of compound **6**.

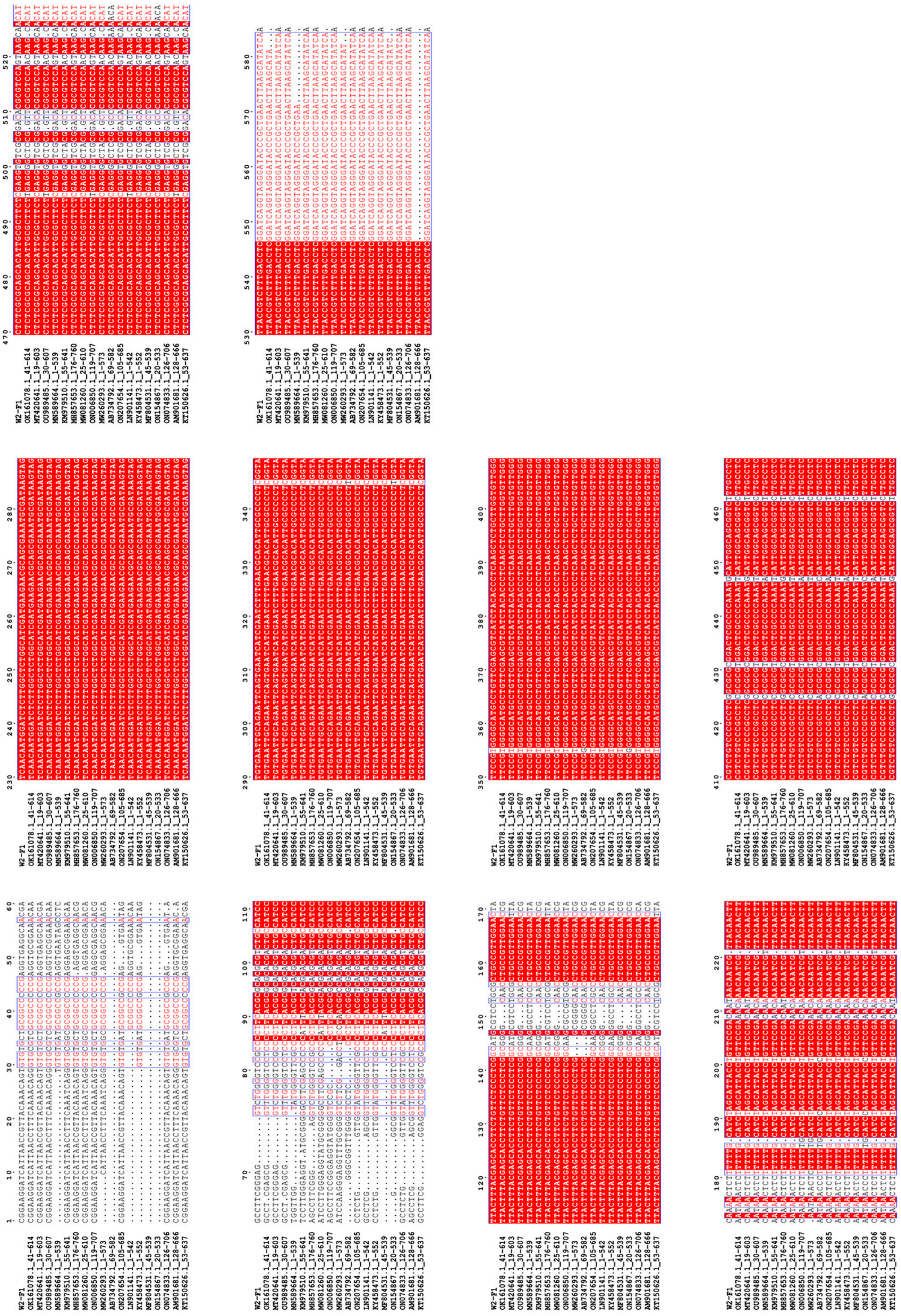

Figure S9. Sequence comparison results of strain W2-F1.

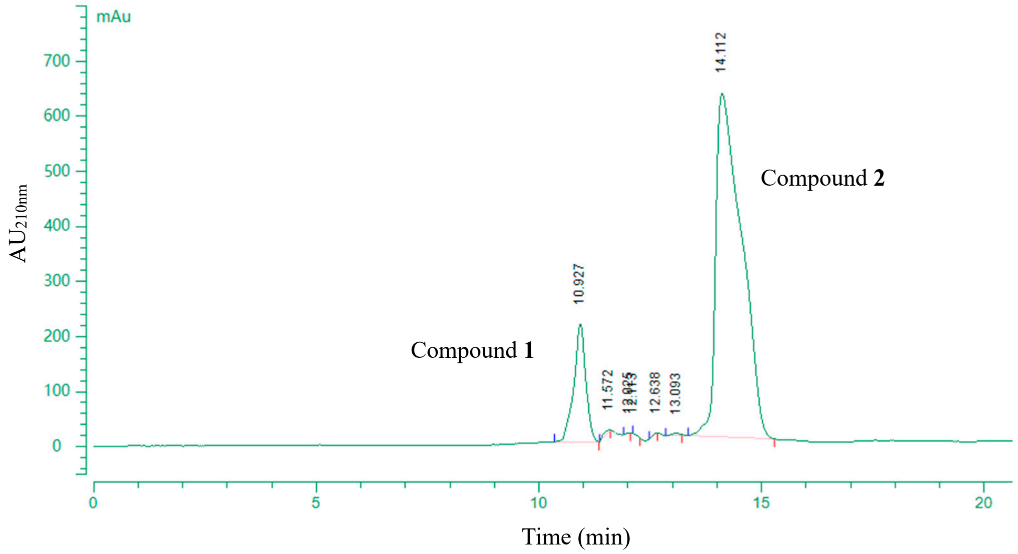

Figure S10. Preparation liquid phase chromatogram of compounds **1** and **2**.

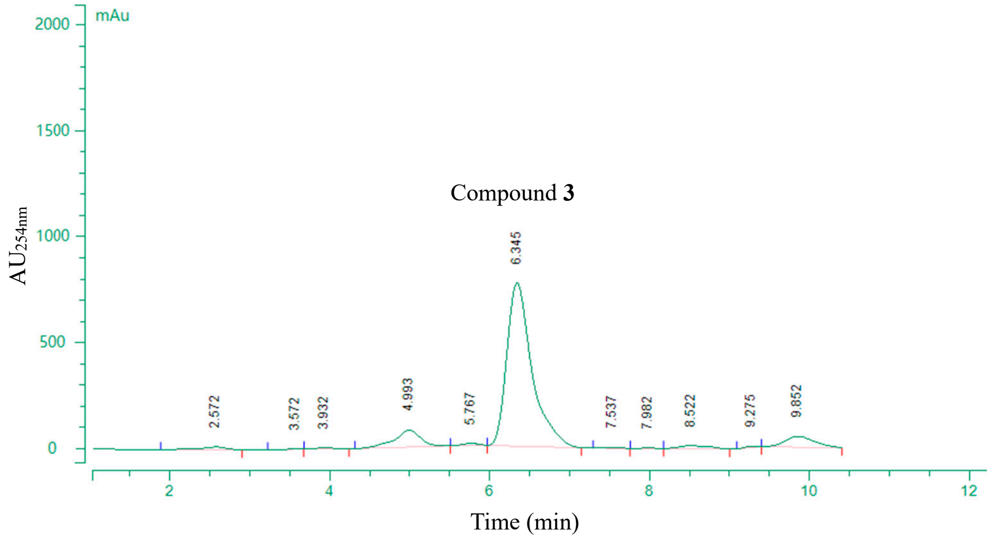

Figure S11. Preparation liquid phase chromatogram of compound **3**.

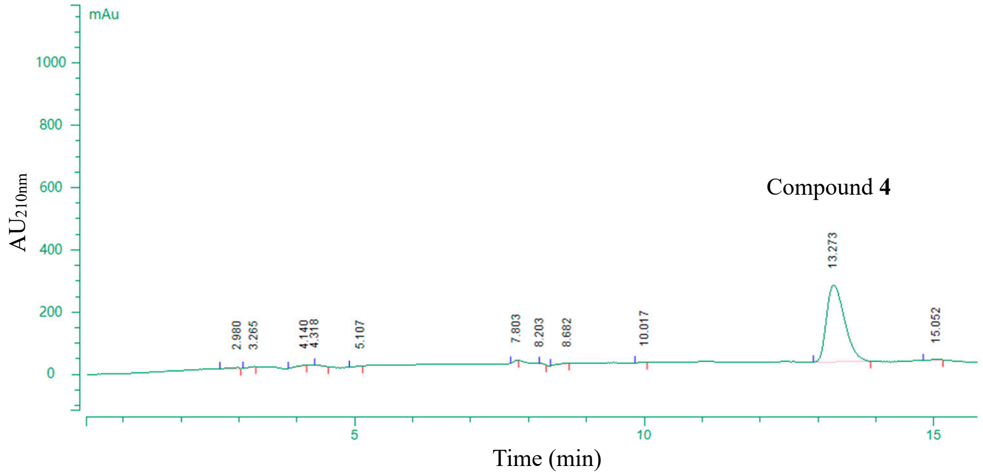

Figure S12. Preparation liquid phase chromatogram of compound **4**.

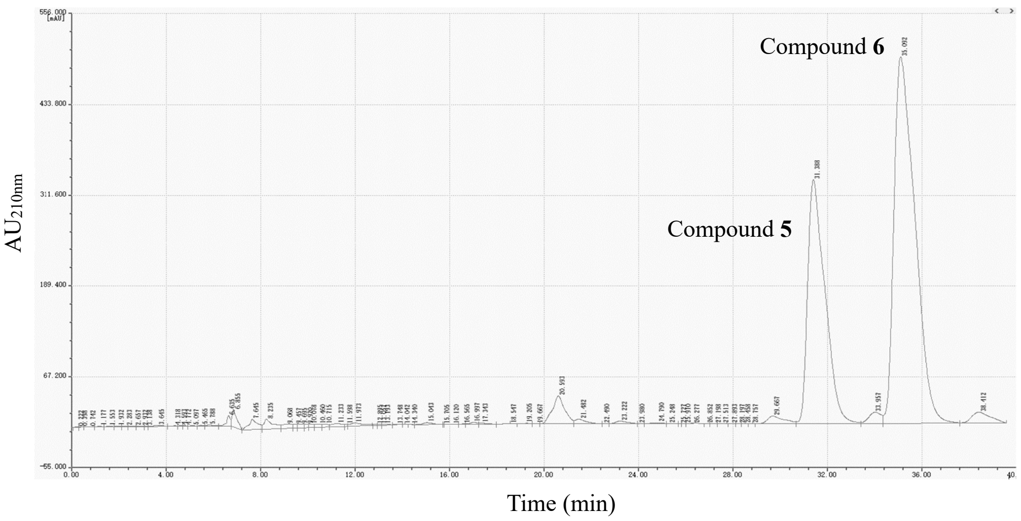

Figure S13. Preparation liquid phase chromatogram of compounds **5** and **6**.

Table S1. The chemical shifts of each hydrogen bearing chiral carbon.

	The chemical shifts of each hydrogen bearing chiral carbon		
	The NMR data from reported papers		Our isolated compounds
cyclo-(Val-Pro)	L-Pro: 3.94 (s) (CDCl3)<br>Val: 4.08 (t, *J* = 7.5) [1]	D-Pro: 4.09 (dt) (CDCl3)<br>D-Val: 3.94 (br.s) [2]	Pro: 4.03 (s, 1H)<br>Val: 4.20 (t, *J* = 7.2 Hz, 1H)
cyclo-(Ile-Pro)	L-Pro: 4.10 (br,s) (CDCl3)<br>L-Ile: 4.22 (t, *J* = 7.1 Hz) [3]	D-Pro: 4.07 (t) (CDCl3)<br>D-Ile: 3.96 (br.s) [2]	Pro: 4.07 (s, H-4, 1H)<br>Ile: 4.19 (t, *J* = 7.0 Hz, 1H)
cyclo-(Leu-Pro)	L-Pro: 4.15 (br.s) (MeOD)<br>L-Leu: 4.29 (t, *J* = 7.1) [4]	D-Pro: 4.27 (t, *J* = 7.5) (MeOD)<br>D-Leu: 4.13-4.16 (m) [5]	Pro: 4.12 (dd, *J* = 6.8, 3.7 Hz, 1H)<br>Leu: 4.25 (t, *J* = 7.1 Hz, 1H)

References

1. Campbell, J.; Lin, Q.; Geske, G. D.; Blackwell, H. E. New and Unexpected Insights into the Modulation of LuxR-Type Quorum Sensing by Cyclic Dipeptides. *ACS Chem. Bio.* **2009**, *4*, 1051-1059. https://doi.org/10.1021/cb900165y.
2. Fdhila, F.; Vázquez, V.; Sánchez, J. L.; Riguera, R., DD-Diketopiperazines: Antibiotics Active against *Vibrio anguillarum* Isolated from Marine Bacteria Associated with Cultures of *Pecten maximus*. *J. Nat. Prod.* **2003,** *66*, 1299-1301. https://doi.org/10.1021/np030233e.
3. Song, S.; Fu, S.; Sun, X.; Li, P.; Wu, J. e.; Dong, T.; He, F.; Deng, Y. Identification of cyclic dipeptides from *Escherichia coli* as new antimicrobial agents against *Ralstonia Solanacearum*. *Molecules* **2018**, *23*, 214. https://doi.org/10.3390/molecules23010214.
4. Yang, B.; Dong, J.; Zhou, X.; Yang, X.; Lee, K.J.; Wang, L.; Zhang, S.; Liu, Y. Proline-containing dipeptides from a marine sponge of a *Callyspongia* Species. *Helv. Chim. Acta* **2009**, *92*, 1112–1117. https://doi.org/10.1002/hlca.200800422.
5. Han, Y.; Li, Y. Y.; Shen, Y.; Li, J.; Li, W. J.; Shen, Y. M., Oxoprothracarcin, a novel pyrrolo[1,4]benzodiazepine antibiotic from marine *Streptomyces* sp. M10946. *Drug Discov. Ther.* **2013**, *7*, 243-7. https://doi.org/10.5582/ddt.2013.v7.6.243.
